# Construction of a Three‐Dimensional Preventive Intervention Model for Nurses’ Job Burnout: Integration of Multiple Theories and Pilot Verification in Obstetrics and Gynecology Nurses

**DOI:** 10.1155/jonm/4889932

**Published:** 2026-06-02

**Authors:** Huiyan Wang, Yucui Meng, Yuxia Mu, Ying Feng, Yuan Zhang, Yongmian Yang, Haibin Zhang

**Affiliations:** ^1^ The Fourth Hospital of Shijiazhuang, No. 16 Tangu North Street Chang’an District, Shijiazhuang City, Hebei, 050000, China

**Keywords:** job burnout, nurse, organizational management, patient safety, three-dimensional preventive intervention model

## Abstract

Given the current severe global shortage of nursing staff, nurse burnout has become a hidden risk threatening patient safety. Alleviating nurse burnout and ensuring patient safety are therefore the core tasks of current clinical nursing management. To address the inadequacies of existing nursing interventions, which are mostly one‐dimensional and lack an integrated framework, this study integrates the core logic of the Conservation of Resources Theory, Job Demands–Resources Model, Effort–Reward Imbalance Model, Role Stress Theory, and Social Cognitive Theory. Combined with the definition of burnout dimensions in the Maslach Burnout Inventory, a “Three‐Dimensional Preventive Intervention Model” suitable for nurses is constructed from the perspective of intervention implementation. The model consists of a framework structure covering three levels: organizational management—individual nurse—career significance. Specifically, the organizational management focuses on dynamic adjustment of workload to control resource depletion; the individual nurse provides psychological resource support to enhance resource reserves; and career significance activates professional value to achieve resource appreciation. Additionally, the model fully considers key influencing factors related to each dimension, establishing an operable and flexible intervention system for practical clinical settings. To verify the model’s feasibility, a preliminary effectiveness validation was conducted in the obstetrics and gynecology department, where nurse burnout is highly prevalent. The results show that this model can provide medical institutions with a standardized general framework for burnout prevention measures at the organizational level. It also allows for dynamic adjustment of intervention measures in a modular manner according to the characteristics of different clinical departments and enables targeted optimization of key priorities across different dimensions. Overall, the model offers replicable and promotable experiences for effective burnout prevention in clinical departments in the future.

## 1. Introduction

Nurse burnout has long been a critical practical issue of global concern, and its importance has become increasingly prominent amid the growing shortage of nurses worldwide [1]. As an “occupational phenomenon” causing both physical and psychological harm, it has been defined and listed as a specific disease in the World Health Organization’s (WHO) International Classification of Diseases, 11th Revision [2], and is categorized as a stress‐induced injury syndrome. Data reports at home and abroad show that the current prevalence of burnout among 288,000 nurses across 32 countries and regions worldwide is 30.7%–0.2 times higher than that of the general population. Notably, the incidence is even higher among nurses working in intensive care units (ICUs), pediatric departments, and obstetrics and gynecology departments [3]. The situation is more severe in China, where the incidence of nurse burnout ranges from 47.97% to 80% [4]. Among Chinese nurses, the rate of severe emotional exhaustion reaches 46%, and the rate of severe personal accomplishment deficiency is nearly 50%—far exceeding the global average [5].

Nurse burnout not only impairs nurses’ physical and mental health but also poses a systemic threat to patient safety and the quality of nursing care. Chronic burnout can lead to a 12% reduction in the gray matter density of nurses’ prefrontal cortex [6]—a brain region associated with executive attention and risk decision‐making. The cognitive decline caused by such structural changes may result in erroneous judgments, simplified safety verification procedures, and reduced risk identification capabilities. These issues further contribute to a series of adverse events, such as patient falls and medication errors, thereby increasing patient safety risks [7].

Although existing classical theories provide a basis for explaining the mechanism of burnout, they have significant practical limitations. The Conservation of Resources (COR) Theory [7] centers on the core logic of “resource depletion > replenishment,” while the Job Demands–Resources (JD–R) Model [8] aims to achieve a balanced state between “workload and resource supply.” On the one hand, neither theory can form a reasonable practical application path for organizational‐management and organizational‐scenario linkage mechanisms. On the other hand, the theories mostly remain at the level of explaining mechanisms and fail to define actionable intervention paths in specific clinical practices. They offer no specific guidance for organizational‐level methods (e.g., “inter‐departmental resource allocation” and “activation of professional meaning”), making it difficult for medical institutions to develop targeted and effective prevention programs based on them.

## 2. Review of Existing Theoretical Models

In the practice of developing preventive interventions for nurse burnout, the research team found that when applying common burnout theoretical models, the unique occupational characteristics of nurses—high emotional investment, fluctuating workload, and intertwined multiple roles—are often overlooked. This easily leads to deviations from the core goal of interventions. Specifically, the team identified obvious limitations in several commonly used burnout models: first, some models (e.g., COR [7], Effort–Reward Imbalance [ERI] Model [9]) overemphasize resource depletion or the “input‐output” perspective; while they can explain the mechanism of burnout, they fail to comprehensively assess the dynamic interaction between individual nurses and the nursing organizational system. Second, another model (JD–R) focuses excessively on static dimensions of the work environment and does not account for the need for timely interventions to address workload fluctuations (e.g., peak delivery periods in obstetrics and gynecology departments) [8]. Third, some models involving multilevel interactions (e.g., Role Stress Theory [RST] and Social Cognitive Theory [SCT]) [10, 11] neglect occupation‐specific factors influencing nurse burnout (e.g., compassion fatigue), resulting in a disconnect between theory and clinical nursing practice. Finally, no existing model identifies “professional meaning” as an independent intervention dimension; as an effective approach to alleviating nurses’ “low personal accomplishment,” professional meaning is currently categorized under “resource replenishment” or “work resources” due to insufficient attention. Below is a detailed explanation and interpretation of each model.

COR is one of the most widely used important theories in burnout research, and its core logic holds that “mental health depends on the acquisition and maintenance of psychological resources, and the depletion of psychological resources exerts a greater impact than resource gain.” This theory can explain burnout triggers such as “mental energy exhaustion from prolonged overtime work” and “loss of social resources due to lack of emotional support from colleagues,” but it also has certain limitations. On the one hand, it emphasizes the “resource depletion–resource replenishment” cycle but ignores “organizational management dynamic adjustment mechanisms,” that is, it overlooks regulatory measures such as workload redistribution among departments based on patient volume to reduce resource depletion at the source. On the other hand, it classifies “Career Significance” under “psychological resources,” overlooking the “implicitness of meaning” experienced by nurses (e.g., inability to perceive work value from repetitive basic care), which disperses intervention focus at career significance. For example, for obstetrics and gynecology nurses, even if psychological counseling helps them regain emotional resources, they may still experience persistent burnout if their work is not infused with professional meaning.

For COR, JD–R is an important supplement; on the basis of COR Theory, it further divides the work environment into job demands and job resources and emphasizes that a dual intervention approach—“reducing demands + increasing resources”—is an effective improvement strategy [8]. However, there are two major problems in its application among nurses: First, the definitions of job demands and job resources in the model are fixed, failing to adapt to the fluctuating workload of nurses. For instance, it does not account for the need to temporarily increase work resources during peak periods in obstetrics and gynecology departments, making the “fixed reduction of job demands” impractical in clinical settings. Second, it does not recognize “professional meaning” as an independent value dimension, instead classifying it as a subcategory of “intrinsic work resources.” This overlooks the fact that nurses require targeted “meaning activation” interventions due to their high emotional investment and delayed feedback from professional meaning (e.g., long patient recovery cycles). Compared with COR, while the JD–R covers the organizational level, it lacks dynamic and occupation‐specific intervention design.

The ERI Model is derived from the perspective of social exchange theory; it posits that prolonged imbalance between effort and reward is the primary cause of burnout, with “overcommitment” acting as a moderating factor [12]. The advantage of this model is that it highlights the importance of “reward mechanisms” for organizational management, but its limitations are also obvious. On the one hand, the model’s reward dimension only includes material or positional rewards (e.g., professional recognition, promotion opportunities) and excludes emotional rewards that nurses value highly (e.g., patient gratitude, colleague recognition)—key sources of perceived work value for nurses. Second, it does not consider that role conflicts amplify nurses’ effort; beyond being caregivers, nurses also act as communicators and managers, and their “effort” includes not only time investment but also psychological burden from role transitions. Additionally, the model quantifies “effort” solely as time input, failing to fully measure the actual workload pressure on nurses.

RST is a classic model for explaining role‐related burnout in nurses; it categorizes burnout triggers into three types: role ambiguity, role conflict, and role overload [13]. It accurately captures the role‐specific traits of nursing, but it has two important limitations: First, it does not separately address “psychological resource restoration” within nurses’ role stress; second, it ignores the interaction between professional meaning and role stress. For example, nurses experiencing burnout from “role overload” could partially offset the negative impact of role stress by strengthening “Career Significance” (e.g., clarifying the significance of multirole efforts for patient recovery) to enhance their psychological resilience, but this connection is not incorporated into the theoretical framework.

SCT holds that burnout is determined by the interaction of “individual‐environment‐behavior,” with self‐efficacy and observational learning as core influencing factors [11]. This model has the advantage of linking individual nurses to the organizational system, but it has limitations when applied to nurses: first, it overlooks the uniqueness of nurses’ interactions with service recipients, that is, nurses’ self‐efficacy stems not only from peer role models but also from positive patient feedback; at the same time, the model generalizes nurse–patient interaction factors as part of the “environment,” ignoring the professional significance of patient feedback for nurses’ self‐efficacy. Second, it neglects the impact of nurses’ compassion fatigue on self‐regulation; prolonged emotional empathy with patients reduces nurses’ emotional regulation capacity, a characteristic not addressed by the “self‐regulation” concept in the model, which inevitably leads to insufficient targeting of corresponding interventions.

MBI was the first to conceptualize burnout as a three‐dimensional structure—emotional exhaustion, depersonalization, and reduced personal accomplishment—and it is one of the core frameworks for burnout assessment [14]. However, it is essentially a descriptive model rather than an intervention‐oriented model, with the following limitations: ① it only focuses on the “symptomatic dimensions” of burnout and ignores the influence of “organizational management factors” on these dimensions, making it unsuitable for guiding organizational management interventions and ② it fails to establish a causal relationship between “professional meaning” and “reduced personal accomplishment”—nurses’ “reduced personal accomplishment” is mainly caused by the lack of “professional meaning,” but the model merely classifies it as a burnout dimension and provides no corresponding “meaning enhancement” interventions, thus offering no help in addressing the root cause of the issue.

**FIGURE 1:**
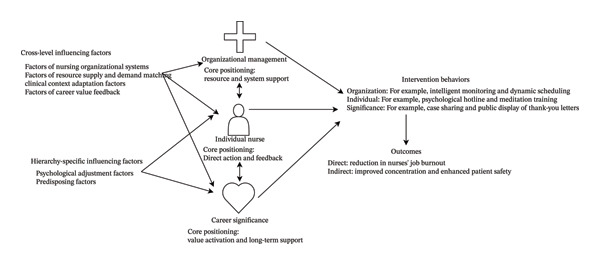
Three‐dimensional preventive intervention model for nurse occupational burnout (Note: Influencing factors are detailed in Table 2).

In summary, based on an analysis of the limitations of existing theoretical models, this study has intuitively summarized the coverage of each model across the three dimensions and clearly demonstrated the absence of coverage and limitations of each model in the professional meaning dimension (see Table 1), thereby providing specific theoretical references for the development of the three‐dimensional model. Furthermore, by integrating the core tenets of multiple models, this study has designed a three‐dimensional preventive intervention model encompassing organizational management, individual nurse, and career significance (see Figure 1), which has been applied to the conceptualization, development, and testing of interventions for nurse burnout.

**TABLE 1:** Specific levels related to organizational management, individual nurses, and/or career significance in the theoretical models evaluated when constructing the three‐dimensional preventive intervention model.

Theoretical model	Organizational management	Individual nurse	Career significance
Conservation of Resources (COR) Theory	—	√	—
Job Demands–Resources (JD–R) Model	√a	√	—
Effort–Reward Imbalance (ERI) Model	√	—	√b
Role Stress Theory	√	√	—
Social Cognitive Theory (SCT)	√	—	√
Maslach Burnout Inventory (MBI)	—	√	—

*Note:* (a) The organizational management level of this model focuses on the classification of static job demands and resources, with no inclusion of dynamic adjustment mechanisms. (b) The career significance of this model is implicit in “career recognition” (a component of “rewards”) and has not been established as an independent dimension.

## 3. Construction Method of the Three‐Dimensional Preventive Intervention Model

This model is built on previous studies of influencing factors and interventions for nurse burnout, draws on key factors from classic theories such as COR and the JD–R Model, and supplements new elements such as “dynamic adjustment” and “career significance” to address the limitations of existing theories. The following sections will elaborate on the construction process of the three‐dimensional preventive intervention model, explain how it compensates for the shortcomings of other burnout theoretical models, and describe how it can be adapted to nurse groups in different clinical departments.

### 3.1. Components of the Model at the Individual Nurse and Organizational Management Levels

JD–R can effectively connect solutions for influencing factors existing simultaneously at both the organizational management level and the individual nurse level. From another theoretical perspective, although the COR is dominated by the model of individual nurses’ resource consumption, current practices have demonstrated that organizational‐level initiatives to mobilize resources, improve nurses’ workload, and alleviate individual resource depletion have, to a certain extent, integrated organizational management and individual nurse management to address the issue of “resource supply‐demand balance” between the two. For example, a hospital’s neurosurgery department implemented flexible scheduling and “temporary rest breaks” for on‐duty nurses. A comparison of nurses’ work status before and after the intervention showed that after the implementation of flexible scheduling, nurses’ emotional exhaustion scores decreased from 68.52 ± 8.36 to 42.15 ± 7.89 and depersonalization scores decreased from 56.38 ± 7.54 to 31.26 ± 6.92 (both *p*  <  0.01), with a corresponding reduction in work pressure [15]. In fact, this is a responsive organizational intervention targeting individual nurses based on their workload, with favorable outcomes.

In addition, in interventions for emergency department nurses, “perceived organizational resource support” at the organizational management level may play a role in repairing nurses’ individual psychological resources [16]. Emergency department nurses often face a large number of emergency tasks and highly complex patient conditions. If hospitals only emphasize the speed of emergency treatment without allocating relevant psychological support resources, nursing staff will re‐experience negative emotions due to the need to continue engaging in emergency rescue work, even after participating in psychological intervention courses. Furthermore, nurses’ perception of organizational resources will further affect the effectiveness of interventions. When nurses perceive that “the hospital values work output more than employee health,” it will lead to poor recovery of their psychological resources, as well as increased psychological distress and low job satisfaction [17]. Therefore, when hospitals promote “perceived resource support” among nursing staff, some individual‐level interventions may yield suboptimal results.

Another major advantage of COR Theory is its ability to examine and act on both the organization and nurses themselves while evaluating key factors influencing both parties. For example, regarding nurse burnout, the most direct measure derived from this theory is to incorporate the two key points of “resource depletion compensation” into both organizational and individual nurse levels, rather than relying solely on nurses to “actively seek resource replenishment” on their own.

In view of this, when formulating strategies at the organizational level, it is necessary to consider nurses’ “individual resource depletion” and the “resource demand” in the workplace. The specific interaction mode between the organization and individuals can be designed based on the “job demands–resources” two‐way model in the JD–R. Moreover, the interaction between the two is dynamic, which requires distinguishing between “static resource allocation” and “dynamic workload adjustment.” Both should be adjusted according to actual work intensity and included in the “organization‐individual” model.

### 3.2. Components of the Career Significance Level and Significance of Its Inclusion

As mentioned earlier, interventions at the organizational management and individual nurse have been discussed, but there remains a critical gap in current nurse burnout intervention models: insufficient attention to “activation of professional meaning.” This issue will be further analyzed below.

MBI, a core tool for assessing burnout, identifies “reduced personal accomplishment” as one of the key dimensions of nurse burnout. Drawing on SCT, which posits a correlation between “self‐efficacy and professional value” [18], this framework suggests that nurses’ sense of professional meaning may diminish due to “reduced personal accomplishment.” However, most existing intervention models pay little attention to activation of “Career Significance”. This study endorses this perspective and argues that attention should be directed to the dimension of professional meaning, with activation of “Career Significance” established as an independent entry point. Only in this way can factors related to professional meaning be optimized to the greatest extent. From the perspective of the three‐dimensional structure, career significance holds a unique value in this dimension: It is influenced not only by nurses’ individual “self‐efficacy” but also by organizational “value feedback,” positive patient interactions, and other factors.

Multiple authoritative practical studies in nursing have provided evidence for the significance of professional meaning. One study demonstrated that implementing professional identity development programs—including guidance on professional values, demonstration of professional achievements, and reinforcement of professional meaning—reduced nurses’ burnout, with scores for personal accomplishment increasing by 20% [19]. Another study focused on reforms to nursing professional models in a hospital, which included recognition of nurses’ professional achievements (e.g., research result‐sharing sessions, awards for excellent case studies) and mechanisms to encourage teamwork. An 18‐month follow‐up survey showed a significant reduction in nurses’ total burnout scores (*p*  <  0.01), with a 23% increase in scores for the personal accomplishment dimension and a 15‐point rise in job satisfaction scores (out of 100) [20]. These findings all illustrate the role of professional meaning in alleviating burnout.

The above research highlights key areas of activity for nurse burnout intervention: patient value feedback, professional honor incentives, and the display and sharing of work outcomes. By selecting appropriate activity formats based on the characteristics of departmental nursing work and fully considering the work scenarios and specific circumstances of nursing staff, burnout interventions can ultimately achieve the goals of alleviating burnout and enhancing personal accomplishment.

Based on the above model construction logic, this study designed targeted modular intervention measures combined with the clinical characteristics of obstetrics and gynecology and conducted a preliminary single‐center empirical verification. The specific verification process, implementation details, and key results will be elaborated in Section 4.2.

## 4. Research Subjects and Methods

### 4.1. Research Subjects

A convenience sampling method was used to select 50 nurses from the Department of Obstetrics and Gynecology at the Fourth Hospital of Shijiazhuang, Hebei Province, from March 2025 to June 2025. A single‐center, pre–post pilot study was conducted. All participants were registered nurses with 1–20 years of working experience.

#### 4.1.1. Inclusion Criteria

1. Employed continuously for more than 6 months;
2. Scores on the Maslach Burnout Inventory (MBI) meeting the cutoff criteria: emotional exhaustion ≥ 27, depersonalization ≥ 8, and personal accomplishment ≤ 24;
3. Voluntary participation in this study;
4. Clear verbal communication ability.

#### 4.1.2. Exclusion Criterion

Pregnant or lactating nurses.

This study was approved by the Ethics Committee of the Fourth Hospital of Shijiazhuang (approval number: 20250122). All participants provided written informed consent. All procedures were performed in accordance with the ethical standards of the Declaration of Helsinki and the STROBE guidelines for observational studies.

### 4.2. Methods

#### 4.2.1. Research Tools

##### 4.2.1.1. General Information Questionnaire

A self‐designed questionnaire was used to collect demographic and occupational data, including gender, age, working years, educational background, marital status, professional title, night shift status, monthly income, monthly number of night shifts, and monthly overtime hours (Supporting Information S1).

##### 4.2.1.2. MBI

The MBI developed by Maslach et al. [14] was adopted. The scale consists of 22 items covering three dimensions: emotional exhaustion (9 items), depersonalization (5 items), and personal accomplishment (8 items). Each item is rated on a 7‐point Likert scale ranging from 0 to 6, including 14 positive items and 8 reverse‐scored items. The total score ranges from 0 to 132. Higher scores for emotional exhaustion and depersonalization indicate more severe burnout, while lower scores for personal accomplishment indicate more severe burnout. The cutoff values for burnout are emotional exhaustion ≥ 27, depersonalization ≥ 8, and personal accomplishment ≤ 24. The Cronbach’s *α* coefficient of this scale was 0.871.

##### 4.2.1.3. Schulte Grid Attention Assessment

The Schulte Grid test was designed by neurologist Schulte. A standard 5 × 5 grid with randomly arranged digits is used; participants read the digits in ascending order as quickly as possible. Faster completion reflects better attention. This method is widely recognized as a simple, direct, and valid tool for attention assessment [21] and has been used in auxiliary diagnosis and efficacy evaluation of attention function [22]. For adults aged 18 years and above, excellent performance is approximately 8 s, and average performance is approximately 20 s. In this study, a computerized 5 × 5 Schulte grid was used to measure changes in attention reaction time before and after the intervention.

#### 4.2.2. Data Collection Methods

Data were collected using an online questionnaire (Wenjuanxing) and semistructured interviews. With approval from the nursing department:

1. Questionnaires were administered before intervention and 3 months after intervention. Participants were provided with standardized instructions and completed the questionnaires independently. A total of 53 questionnaires were distributed, and 50 valid questionnaires were recovered, with an effective response rate of 94.34%.
2. A computerized 5 × 5 Schulte grid test was conducted before intervention and 3 months after intervention to measure attention reaction time. All 50 participants completed the test, with a participation rate of 100%.
3. Three months after intervention, semistructured interviews were conducted to explore participants’ subjective experience, including intervention perceptions, burnout improvement, changes in attention, and suggestions for model optimization.

## 5. Statistical Analysis

All statistical analyses were performed using SPSS 26.0 software. Measurement data are presented as mean ± standard deviation ($\overset{¯}{x}$ ± *s*). Differences before and after intervention were analyzed using the paired *t*‐test. Enumeration data are presented as cases and percentages (n, %). A *p* value < 0.05 was considered statistically significant.

## 6. Results

### 6.1. The Three‐Dimensional Preventive Intervention Model

In the three‐dimensional preventive intervention model (Figure 1), influencing factors at the organizational management, individual nurse, and career significance levels are presented independently. Some factors (nursing organizational systems, resource supply‐demand matching, clinical context adaptation, and professional value feedback) apply to all three levels, while psychological adjustment and predisposing factors are exclusive to the individual nurse and career significance levels. Table 2 lists examples of each factor and related implementation considerations.

**TABLE 2:** Factors influencing the organizational management, individual nurse, and career significance in the three‐dimensional preventive intervention model and related implementation considerations.

Core factors	Organizational management	Individual nurse	Career significance
Nursing organization system	Dynamic scheduling mechanism	Scheduling adaptability	The embodiment of professional values in the system
	Nurse workload monitoring system	Perception of the rationality of task allocation	Organizational resource support
	Backup nurse support plan	Perception of fairness in performance evaluation	professional meaning activities
	Standards for the division of nursing tasks	Satisfaction with organizational support	
	Department performance incentive rules (including burnout prevention and control indicators)		

Resource supply and demand matching	Nursing staffing	Personal energy reserve	Career development resources
	Psychological support resources	Reserve of psychological resources	Meaning perception resources
	Vocational training resources	Skill resource reserve	
	Material and equipment support		

Clinical situation adaptation	Department work intensity fluctuation response plan	Clinical situation response ability	Meaning mining in different clinical contexts
	Mechanism for manpower allocation in emergency situations	Ability to control emotions in high‐pressure situations	
	Inter‐departmental collaboration process	Nursing fit for special patients	

Professional value feedback	Public notice of patients’ thank‐you letters	Frequency of receiving positive feedback from patients	Direct contact with patients′ rehabilitation cases
	Monthly report on nursing outcomes	The degree of recognition of nursing work by colleagues/leaders	Cognitive understanding of the social value of professional contributions
	Nursing professional honor system	Self‐perception of personal care outcomes	
	Quality improvement achievement sharing session in nursing	Degree of achievement of career goals	
	Family member satisfaction feedback mechanism		

Psychological adjustment	—	Degree of emotional exhaustion	The influence of mental state on the perception of meaning
		Level of psychological resilience	The feedback of a sense of meaning on psychological adjustment
		Self‐efficacy	
		Empathy fatigue prevention and control ability	

Predisposing factors	—	Demographic characteristics	Career values
		Professional belief	Tendency toward meaning perception
		Past negative experiences	
		Personal health habits	

The core factors in Table 2 are derived from the COR, the JD–R, and the three dimensions of the MBI. The detailed considerations are summarized based on clinical practices related to nurse burnout intervention and literature on nursing management. The three‐dimensional preventive intervention model is flexible enough to meet the needs of nurse burnout interventions at different levels. Additionally, the composition and implementation points of each factor can be considered in an integrated manner, but not all factors or implementation points need to be included. For example, interventions for obstetrics and gynecology nurses should pay more attention to the “clinical context adaptation factor” to avoid peak delivery periods, while interventions for oncology nurses should focus more on the “professional value feedback factor.”

It is worth emphasizing that the three‐dimensional preventive intervention model can incorporate dimensions and factors that are not covered by conventional burnout theoretical models. For instance, it addresses the underappreciated “individual differences in workload tolerance” among nurses (i.e., nurses of different ages may have different energy reserves) [23]. Furthermore, while theories such as the COR and the JD–R focus on “universal resource replenishment,” they overlook the diverse needs of individual nurses, failing to provide targeted “precision” resource replenishment opportunities. Current research indicates that psychological interventions (as a form of resource replenishment) can alleviate nurses’ “compassion fatigue” but have little impact on “value perception indicators (personal accomplishment, empathy satisfaction)” and lack long‐term effectiveness [24]. This phenomenon arises precisely because organizations do not recognize the independence of the “professional meaning level,” a gap that the Three‐Dimensional Model effectively addresses.

### 6.2. Preliminary Empirical Validation of the Three‐Dimensional Preventive Intervention Model Among Obstetrics and Gynecology Nurses

In the conceptual validation phase of this study, a convenience sampling method was adopted to conduct a single‐center pre–post pilot study among 50 nurses working in the Department of Obstetrics and Gynecology, the Fourth Hospital of Shijiazhuang, Hebei Province, from March 2025 to June 2025. Targeting job burnout, decreased attention, and patient safety risks among nurses, the three‐dimensional preventive intervention model was preliminarily tested. Specific intervention components included smartwatch physiological monitoring, cross‐departmental mobile support, and diversion of noncore work tasks; mindfulness meditation, artificial intelligence mood journaling, occupational burnout emergency kits, and safety narrative workshops; as well as narrative nursing sharing sessions, career value perception dashboards, and safety proposal feedback mechanisms (see Table 3).

**TABLE 3:** Practical application of the three‐dimensional preventive intervention model in nurse burnout intervention.

Level	Practical intervention measures
Organizational management	1. Establish an intelligent management system with workload monitoring and early warning functions.
	2. Appoint dedicated leaders for nurse burnout prevention and control.
	3. Build an inter‐unit mobile nurse support pool.
	4. Implement the Assessment, Feedback, Incentive, and Exchange (AFIX) program to dynamically manage nurses’ workload and burnout.
	5. Coordinate staff to identify nurses in need of intervention; ensure continuous burnout prevention and control support throughout all clinical work processes.
	6. Develop a professional honor system (e.g., “Nursing Star” selection) and a platform for displaying nursing achievements.

Individual nurse	1. Provide training on smart device (e.g., smartwatch) operation and physiological workload monitoring.
	2. Deliver training on psychological support measures (e.g., daily meditation, burnout emergency kits, psychological hotlines).
	3. Conduct skill training (e.g., emergency response, nurse–patient communication skills).
	4. Provide guidance on career significance perception (e.g., organizing nurses to participate in patient recovery sharing sessions).

Career significance	1. Establish positive patient feedback channels (e.g., public display of thank‐you letters, follow‐up sharing of recovery cases).
	2. Launch nursing career narrative activities to encourage nurses to share professional growth and value realization stories.
	3. Establish a mechanism for disseminating the social value of nursing work; promote nursing contributions through media and other channels.

A combination of the MBI, Schulte Grid test, and semistructured interviews was used to comprehensively evaluate intervention effects from three dimensions: burnout level, attention state, and subjective experience. The results showed that although patient safety outcome measures were not included in this pilot, the average implementation rate of interventions reached above 87%. After intervention, nurses’ scores for emotional exhaustion and depersonalization were significantly decreased (*p*  <  0.05), scores for personal accomplishment were significantly increased (*p*  <  0.05), and reaction time on the Schulte Grid test was reduced by 31.73% compared with baseline (*p*  <  0.05). Participants reported that the intervention could alleviate occupational burnout and improve concentration. Based on the collected optimization suggestions, the research team will further refine this multicomponent intervention program for nurse burnout. Detailed implementation data and statistical analysis results are presented in Supporting Information S1 and S2.

## 7. Discussion

### 7.1. Application of the Three‐Dimensional Preventive Intervention Model in Nurses’ Job Burnout

The greatest value of the three‐dimensional preventive intervention model lies in providing specific, implementable, and evaluable measures for the implementation and assessment of nurse burnout interventions. Based on the three‐dimensional preventive intervention model (Figure 1, Table 2), key intervention measures applicable to nurse burnout were identified through literature review and team research (Table 3). However, these measures do not cover all possibilities, including (a) optimal monitoring practices for identifying nurses’ physiological and psychological workload overload; (b) evaluation and feedback on the effectiveness of individual nurse interventions; (c) appointment of burnout prevention leaders in medical institutions; (d) continuous promotion of and support for burnout prevention behaviors throughout the entire clinical workflow; (e) provision of training on burnout prevention for healthcare staff; and (f) customization of intervention programs for nurse groups with different characteristics (e.g., integrating factors such as age, professional title, frequency of night shifts, psychological counseling, and human resource support).

These examples can guide the implementation of interventions under the framework of the three‐dimensional preventive intervention model and demonstrate the model’s flexibility in adapting measures to different nurse groups and clinical scenarios. For instance, in emergency departments with high work intensity and frequent night shifts, the frequency of intelligent workload monitoring and rapid‐response human resource support can be increased; in pediatric departments with high demands for doctor–patient communication and heavy emotional labor, the richness and privacy of psychological support measures can be enhanced. Additionally, strong support from the organizational management level (e.g., vigorous promotion by prevention leaders and orderly operation of support pools) and active participation of individual nurses in various interventions remain essential.

### 7.2. Evaluation of Intervention Measures Using the Three‐Dimensional Preventive Intervention Model

By addressing various factors contributing to nurses′ job burnout through the three dimensions—organizational management, individual nurse, and career significance (Figure 1)—and incorporating key influencing factors (Table 2), a clear pathway can be established for the development of evaluation materials. For example, by integrating the specific intervention activities listed in Table 3 with the influencing factors presented in Table 2, researchers can construct an evaluation framework. This framework enables both process evaluation (for the implementation of intervention measures) and outcome evaluation (for the effectiveness of interventions).

The combination of process evaluation and outcome evaluation serves two key purposes: on one hand, it identifies specific measures that play a critical role in the success of interventions; on the other hand, it uncovers measures that can be eliminated or modified, thereby optimizing the intervention program. Using the three‐dimensional preventive intervention model to design and evaluate intervention measures not only accounts for the multifactorial nature of improving nurses′ job burnout but also supports the development and evaluation of interventions in a modular manner—facilitating the subsequent refinement and optimization of intervention programs.

## 8. Conclusion

This study elaborates on the construction process of the three‐dimensional preventive intervention model for nurses′ job burnout and illustrates the model’s application using the clinical nurse group as an example. Drawing on previous burnout‐related models (COR, JD–R, RST, SCT, and MBI) and research findings from nurse job burnout intervention practices, the three‐dimensional preventive intervention model integrates the core elements of these models to form a comprehensive framework for nurses′ job burnout intervention.

The three‐dimensional preventive intervention model—encompassing its conceptual framework, key considerations for core factors, and specific activities—provides a design, implementation, and evaluation framework for studies assessing the effectiveness of nurses′ job burnout interventions. In terms of design, the model balances practical application with flexibility to adapt to different clinical departments and nurse groups, making it a flexible and adaptable framework suitable for various nurse job burnout intervention activities.

The examples provided in this study are not intended to cover all potential application scenarios of the three‐dimensional preventive intervention model; instead, they aim to offer a framework reference for the model’s application in diverse contexts.

## Author Contributions

Huiyan Wang: writing–review and editing, writing–original draft, visualization, validation, methodology, investigation, formal analysis, and data curation. Yucui Meng: writing–original draft, visualization, validation, supervision, software, methodology, and formal analysis. Yuxia Mu: writing–original draft, visualization, validation, supervision, software, methodology, and formal analysis. Ying Feng: supervision, project administration, investigation, and data curation. Yuan Zhang: investigation and data curation. Yongmian Yang: investigation and data curation. Haibin Zhang: writing–review and editing, validation, supervision, resources, project administration, and conceptualization.

## Funding

This research did not receive any specific grant from funding agencies in the public, commercial, or not‐for‐profit sectors.

## Conflicts of Interest

The authors declare no conflicts of interest.

## Supporting Information

Additional supporting information can be found online in the Supporting Information section.

## Supporting information


**Supporting Information 1** Table S1: Characteristics of participants (*N* = 50). Table S2: Implementation of feasibility indicators for intervention measures of the three‐dimensional preventive intervention model among obstetrics and gynecology nurses (*N* = 50). Table S3: Comparison of Maslach Burnout Inventory dimensions and Schulte grid reaction time before and after intervention (*N* = 50).

## Data Availability

The data that support the findings of this study are available in the supporting information of this article.
