# Supplementary material for: Construction of a Three‐Dimensional Preventive Intervention Model for Nurses’ Job Burnout: Integration of Multiple Theories and Pilot Verification in Obstetrics and Gynecology Nurses
Source: J Nurs Manag. 2026 Jun 2;2026:4889932. doi: 10.1155/jonm/4889932 (PMC13239261; doi:10.1155/jonm/4889932)
Supplement: Supplementary file 1 — Supporting Information 1 Table S1: Characteristics of participants (N = 50). Table S2: Implementation of feasibility indicators for intervention measures of the three‐dimensional preventive intervention model among obstetrics and gynecology nurses (N = 50). Table S3: Comparison of Maslach Burnout Inventory dimensions and Schulte grid reaction time before and after intervention (N = 50). [file JONM-2026-4889932-s001.zip › Table S2_1.docx]

**Table S2 Comparison of Nurses' Occupational Burnout and Attention Indicators Before and After Intervention (N=50)**

Indicator Pre-Intervention Post-Intervention *t* *p*

Emotional Exhaustion 33.87±10.71 14.81±4.21 15.23 < 0.001

Depersonalization 14.51±5.90 6.38±1.97 12.45 < 0.001

Personal Accomplishment 22.67±4.37 31.73±4.45 -10.89 < 0.001

Attention Reaction Time 41.13±10.39 28.08±7.75 21.45 < 0.001
