# Supplementary material for: Construction of a Three‐Dimensional Preventive Intervention Model for Nurses’ Job Burnout: Integration of Multiple Theories and Pilot Verification in Obstetrics and Gynecology Nurses
Source: J Nurs Manag. 2026 Jun 2;2026:4889932. doi: 10.1155/jonm/4889932 (PMC13239261; doi:10.1155/jonm/4889932)
Supplement: Supplementary file 1 — Supporting Information 1 Table S1: Characteristics of participants (N = 50). Table S2: Implementation of feasibility indicators for intervention measures of the three‐dimensional preventive intervention model among obstetrics and gynecology nurses (N = 50). Table S3: Comparison of Maslach Burnout Inventory dimensions and Schulte grid reaction time before and after intervention (N = 50). [file JONM-2026-4889932-s001.zip › Table S2_2.docx]

**Table S2. Comparison of Maslach Burnout Inventory Dimensions and Schulte Grid Reaction Time Before and After Intervention (N=50)**

| **Indicator** | **Pre-Intervention** | **Post-Intervention** | **t** | **p** |
| --- | --- | --- | --- | --- |
| Emotional Exhaustion | 33.87 ± 10.71 | 14.81 ± 4.21 | 15.23 | < 0.001 |
| Depersonalization | 14.51 ± 5.90 | 6.38 ± 1.97 | 12.45 | < 0.001 |
| Personal Accomplishment | 22.67 ± 4.37 | 31.73 ± 4.45 | -10.89 | < 0.001 |
| Attention Reaction Time | 41.13 ± 10.39 | 28.08 ± 7.75 | 21.45 | < 0.001 |
