# Supplementary material for: Construction of a Three‐Dimensional Preventive Intervention Model for Nurses’ Job Burnout: Integration of Multiple Theories and Pilot Verification in Obstetrics and Gynecology Nurses
Source: J Nurs Manag. 2026 Jun 2;2026:4889932. doi: 10.1155/jonm/4889932 (PMC13239261; doi:10.1155/jonm/4889932)
Supplement: Supplementary file 1 — Supporting Information 1 Table S1: Characteristics of participants (N = 50). Table S2: Implementation of feasibility indicators for intervention measures of the three‐dimensional preventive intervention model among obstetrics and gynecology nurses (N = 50). Table S3: Comparison of Maslach Burnout Inventory dimensions and Schulte grid reaction time before and after intervention (N = 50). [file JONM-2026-4889932-s001.zip › Table S1_1.docx]

**Table S1. Implementation of Feasibility Indicators for Intervention Measures of the Three-Dimensional Preventive Intervention Model Among Obstetrics and Gynecology Nurses (N=50)**

| **Intervention Module** | **Process Indicator** | **Indicator Type** | **Evaluation Criterion** | **Result** |
| --- | --- | --- | --- | --- |
| Physiological Load Monitoring | Smartwatch Wearing Rate | Actual Proportion of Eligible Wearers | Rate (%) | 95.20% |
|  | Rest Execution Rate After Alert | Actual Rest Proportion Among Alert Occurrences | Rate (%) | 91.70% |
| Dynamic Manpower Allocation | Response Time for Support Pool Activation | Average Time for Support Staff to Arrive at Post | Minutes (Mean ± SD) | 18.5 ± 5.2 min |
| Work Task Diversion | Nurses' Core Working Hours | Growth Ratio of Core Working Hours | Rate (%) | 25% |
| Psychological Energy Reserves | Daily Meditation Participation Rate | Actual Proportion of Eligible Participants | Rate (%) | 87.30% |
|  | Timeliness Rate of Burnout Emergency Kit Approval | Proportion of Approvals Completed Within 2 Hours | Rate (%) | 96.40% |
|  | 24-Hour Answer Rate of Psychological Hotline | Proportion of Successful Connections Among Incoming Calls | Rate (%) | 98.10% |
| Other Activity-Based Measures | Frequency of Safety Narrative Workshop Implementation | Planned Monthly Frequency | Times per Month | 3 |
|  | Frequency of Narrative Nursing Sharing Session | Planned Monthly Frequency | Times per Month | 12 |
|  | Frequency of Career Meaning Dashboard Display | Planned Display Frequency | Mode | Real-time |
