# Supplementary material for: Construction of a Three‐Dimensional Preventive Intervention Model for Nurses’ Job Burnout: Integration of Multiple Theories and Pilot Verification in Obstetrics and Gynecology Nurses
Source: J Nurs Manag. 2026 Jun 2;2026:4889932. doi: 10.1155/jonm/4889932 (PMC13239261; doi:10.1155/jonm/4889932)
Supplement: Supplementary file 1 — Supporting Information 1 Table S1: Characteristics of participants (N = 50). Table S2: Implementation of feasibility indicators for intervention measures of the three‐dimensional preventive intervention model among obstetrics and gynecology nurses (N = 50). Table S3: Comparison of Maslach Burnout Inventory dimensions and Schulte grid reaction time before and after intervention (N = 50). [file JONM-2026-4889932-s001.zip › Table S1_2.docx]

**Table S1 Implementation of Indicators for the Feasibility of Intervention Measures (N=50)**

Intervention Module Process Indicator Indicator Type Evaluation Criterion Result

Physiological Load Monitoring Smartwatch Wearing Rate Actual Proportion of Eligible Wearers Rate 95.2%

Rest Execution Rate After Alert Actual Rest Proportion Among Alert Occurrences Rate 91.7%

Dynamic Manpower Allocation Response Time for Support Pool Activation Average Time for Support Staff to Arrive at Post Minute 18.5±5.2

Work Task Diversion Nurses' Core Working Hours Growth Ratio of Core Working Hours Rate 25%

Psychological Energy Reserves Daily Meditation Participation Rate Actual Proportion of Eligible Participants Rate 87.3%

Timeliness Rate of Burnout Emergency Kit Approval Proportion of Approvals Completed Within 2 Hours of Submission Rate 96.4%

24-Hour Answer Rate of Psychological Hotline Proportion of Successful Connections Among Incoming Calls Rate 98.1%

Other Activity-Based Measures Frequency of Safety Narrative Workshop Implementation Planned Monthly Frequency Times/Month 3

Frequency of Narrative Nursing Sharing Session Implementation Planned Monthly Frequency Times/Month 12

Frequency of Career Meaning Dashboard Display Planned Monthly Frequency Times/Month Real-time
