# Supplementary material for: Construction of a Three‐Dimensional Preventive Intervention Model for Nurses’ Job Burnout: Integration of Multiple Theories and Pilot Verification in Obstetrics and Gynecology Nurses
Source: J Nurs Manag. 2026 Jun 2;2026:4889932. doi: 10.1155/jonm/4889932 (PMC13239261; doi:10.1155/jonm/4889932)
Supplement: Supplementary file 1 — Supporting Information 1 Table S1: Characteristics of participants (N = 50). Table S2: Implementation of feasibility indicators for intervention measures of the three‐dimensional preventive intervention model among obstetrics and gynecology nurses (N = 50). Table S3: Comparison of Maslach Burnout Inventory dimensions and Schulte grid reaction time before and after intervention (N = 50). [file JONM-2026-4889932-s001.zip › Table S1.doc]

| Item | Cases/Percentage (%) | Mean±SD |
| --- | --- | --- |
| Gender |  |  |
| Male | 7 (14.00) |  |
| Female | 43 (86.00) |  |
| Age (years) |  |  |
| 18-30 | 23 | 27.22±2.17 |
| 31-40 | 17 | 32.59±1.61 |
| 41-50 | 10 | 42.30±1.55 |
| Highest Education |  |  |
| College | 8 (16.00) |  |
| Bachelor's degree | 40 (80.00) |  |
| Master's degree or above | 2 (4.00) |  |
| Marital Status |  |  |
| Single | 19 (38.00) |  |
| Married without children | 5 (10.00) |  |
| Married with children | 26 (52.00) |  |
| Professional Title |  |  |
| Junior | 26 (52.00) |  |
| Intermediate | 22 (44.00) |  |
| Senior | 2 (4.00) |  |
| Years of Service |  |  |
| 1-5 years | 12 | 2.50±1.38 |
| 5-10 years | 22 | 6.50±1.62 |
| 10-20 years | 16 | 15.88±3.59 |
| Monthly Income (RMB) |  |  |
| <5,000 | 12 (24.00) |  |
| 5,000-8,000 | 26 (52.00) |  |
| >8,000 | 12 (24.00) |  |
| Night Shifts |  |  |
| Yes | 32 (64.00) |  |
| No | 18 (36.00) |  |
| Monthly Night Shifts |  |  |
| 0-3 shifts | 19 | 0.95±1.19 |
| 4-6 shifts | 19 | 5.21±0.89 |
| 7-9 shifts | 8 | 7.88±0.33 |
| >10 shifts | 4 | 11.50±0.50 |
| Monthly Overtime Hours |  |  |
| <24 hours | 17 (34.00) |  |
| 24-48 hours | 16 (32.00) |  |
| 48-72 hours | 5 (10.00) |  |
| >72 hours | 12 (24.00) |  |

**Table S1. Characteristics of participants(N=50)**
